# Protective Effect of Anthocyanins against Neurodegenerative Diseases through the Microbial-Intestinal-Brain Axis: A Critical Review

**DOI:** 10.3390/nu15030496

**Published:** 2023-01-18

**Authors:** Hao Zhong, Jie Xu, Mengyu Yang, Muhammad Hussain, Xiaofeng Liu, Fengqin Feng, Rongfa Guan

**Affiliations:** 1 College of Food Science and Technology, Zhejiang University of Technology, Hangzhou 310014, China; 2 College of Biosystems Engineering and Food Science, Zhejiang University, Hangzhou 310058, China

**Keywords:** anthocyanins, gut microbiota, microbiota-gut-brain axis, neurodegenerative disease, neuroprotection

## Abstract

With the increase in human mean age, the prevalence of neurodegenerative diseases (NDs) also rises. This negatively affects mental and physiological health. In recent years, evidence has revealed that anthocyanins could regulate the functioning of the central nervous system (CNS) through the microbiome-gut-brain axis, which provides a new perspective for treating NDs. In this review, the protective effects and mechanisms of anthocyanins against NDs are summarized, especially the interaction between anthocyanins and the intestinal microbiota, and the microbial-intestinal-brain axis system is comprehensively discussed. Moreover, anthocyanins achieve the therapeutic purpose of NDs by regulating intestinal microflora and certain metabolites (protocateic acid, vanillic acid, etc.). In particular, the inhibitory effect of tryptophan metabolism on some neurotransmitters and the induction of blood-brain barrier permeability by butyrate production has a preventive effect on NDs. Overall, it is suggested that microbial-intestinal-brain axis may be a novel mechanism for the protective effect of anthocyanins against NDs.

## 1. Introduction

Recent investigations revealed an average global rise in life expectancy accompanied by the prevalence of neurodegenerative disease (NDs). The occurrence of NDs is mainly in middle and older age, due to which the number of people with diseases such as Alzheimer’s disease (AD), Parkinson’s disease (PD), and Amyotrophic lateral sclerosis (ALS) is expected to rise soon. Despite the increasing prevalence of these diseases, current treatment approaches are mostly relatively conservative, and the treatment of NDs remains challenging. In this regard, polyphenols as multipotent agents have gained much attention. Particularly, anthocyanins have gained significant attention because they relieve many of the basic symptoms of neurodegenerative processes, including direct action on the central nervous system (CNS) and indirect action on the CNS through the gut microbiota. The claim that anthocyanins regulate neurological disorders via the microbiome-gut-brain axis has also been widely concerned.

**Figure 1:**
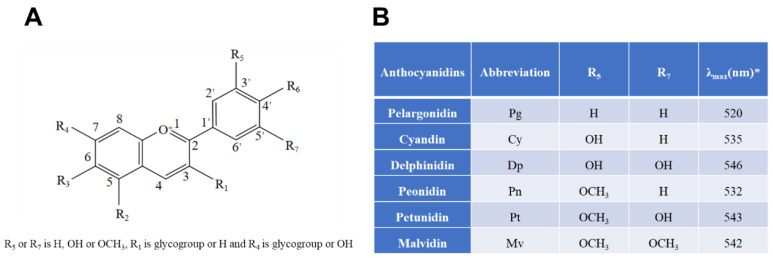
Chemical structures of six common anthocyanidins (flavylium form) and their sources based on current information. (**A**) Basic structure; (**B**), six common anthocyanidins. * indicates that λ_max_ means the wavelength at which a substance has its strongest photon absorption.

Anthocyanins, a class of water-soluble flavonoids, which are natural color pigments found in various colorful plants, such as berry fruits and vegetables, have received extensive attention for their neuroprotective effects in the CNS [1]. Similarly, they have antioxidant, anti-aging [2], anti-inflammatory, antibacterial, vision protection, and other physiological functions, and could also reduce the level of oxidative stress, neuro-inflammation in the body [3], and improve the vitality of microglia. The color of anthocyanins usually changes with pH, and as the pH changes, its color will change from red to different colors such as purple, orange, colorless, and blue. As a result, they are widely used in the food, pharmaceutical, and cosmetic industries [4]. There are more than 20 known anthocyanins in nature, among them cyanidin, delphinidin, malvidin, peonidin, petunidin, and pelargonidin; these six are the most common anthocyanins. The stability of anthocyanins depends on the presence of the B-ring and hydroxyl or methoxy groups in their structure, with anthocyanin-3-glucoside (C3G) being the most widely distributed anthocyanin in nature (Figure 1) [5]. Anthocyanins are considered useful functional food ingredients, conferring various healthy benefits and emerging as promising ingredients for the food industry.

It is assessed that the number of human gut microbiota is made up of about 10 to 100 trillion microorganisms, 11 times that of human body cells, i.e., the body is made up of 10% of our cells and the other 90% is comprised of microbial cells [6]. The gut microbiota is generally classified into harmful, neutral, and beneficial bacteria. Studies have shown that the *Bacteroides* spp. and *Firmicutes* spp. are the most commonly known gut microbes, accounting for approximately 75% of all gut microbiota [7]. The gut microbiota is a complex micro-ecosystem in the human body. It is mutually restricted and interdependent with its host, thus maintaining the homeostasis of the human microecology. If affected by the changes of the host and external environment, microbial homeostasis will be altered, forming destructive physiological combinations and generating pathological combinations, resulting in intestinal microbiota disorders, and even inducing the production of many metabolic diseases, immune diseases, infectious diseases, and some neurological diseases [8,9]. The growing evidence reveals the importance of the gut microbiome in maintaining immune function and metabolic homeostasis in the body [9].

It has long been exhibited that the gastrointestinal tract can be regulated by brain via movement, blood flow, secretion, and absorption, while the gut can also affect the brain’s function and behavior. This two-way communication between the brain and the gut is called the gut-brain axis [10,11]. In addition to coordinating and integrating gastrointestinal functions, it also connects the brain and peripheral functions [12]. Many reactions occur in these processes, such as the activation of immune responses, the protection of intestinal permeability, and the transmission of gastrointestinal-endocrine signals [13]. The gut microbiota is currently recognized as a key regulator of a smooth two-way dialogue between the gut and the brain (gut-brain axis). A few preclinical examinations propose that the microbiome and its genome (microbiome) may assume a critical part in neurodevelopment and NDs. Furthermore, human gut microbiota composition alterations have also been linked to various neuropsychiatric conditions, including depression, autism spectrum disorder (ASD), and PD. Microbiome-gut-brain axis could explain how the gut microbiota mediates the interaction between the gastrointestinal tract and the brain and how the gut microbiota participates in signaling between the nervous, endocrine, and immune systems and the brain [14]. As a result, the microbiome-gut-brain axis has become a potential diagnostic and therapeutic target for many neurological disorders.

There is growing evidence that anthocyanins are therapeutically effective in neurological disorders via the gut microbiota, where the microbiome-gut-brain axis plays a key role. This review focuses on the protective effects of anthocyanins on neurological diseases, their interaction with the gut microbiota, and the possible mechanisms by which anthocyanins and/or their metabolites regulate neurological diseases through the microbial-gut-brain axis.

## 2. Protective Effects of Anthocyanins on Neurodegenerative Diseases

Progressive death of neurons or loss of myelin sheaths can trigger NDs that manifest as brain damage or physical dysfunction, resulting in central and internal nerve deterioration and muscle damage [15]. NDs can be classified into two types: acute and chronic. Acute NDs may cause permanent neurological damage in the short term [16]. Chronic NDs are the main diseases that hinder the quality of life of older adults, such as AD, PD, and ALS.

**Figure 2:**
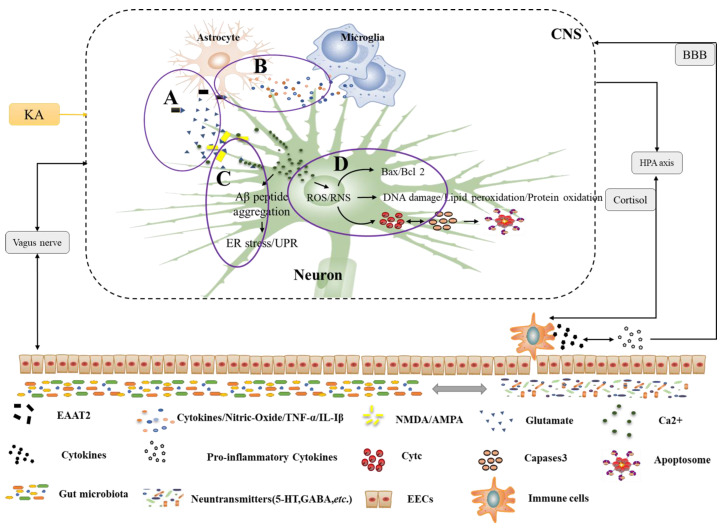
Pathogenesis of NDs under the microbe-gut-brain axis system. A. Excitotoxins. Glutamate is released from neurons and binds to receptors (NMDA, AMPA), along with glutamate uptake by excitatory amino acid transporter 2 (EAAT2) in astrocytes and the action of external factors (e.g., KA) all lead to increased glutamate excitotoxicity, thus promoting Ca^2+^ flow, endoplasmic reticulum stress, and pro-apoptotic signaling cascades. B. Neuroinflammation. Interactions between glial cells predispose to neuroinflammation, especially astrocytes and microglia. They may produce several cytokines and oxidants (e.g., TNF-α and IL-Iβ by astrocytes, nitric oxide, and pro-inflammatory cytokines by microglia) that bind to cell surface death receptors and activate pro-apoptotic signaling cascades. C. Endoplasmic reticulum stress. There are many causes of endoplasmic reticulum stress, mainly abnormal folding of Aβ peptides and activation of the unfolded protein response (UPR). This induces abnormal protein aggregation and increases intracellular calcium efflux, which triggers a pro-apoptotic signaling cascade. D. Oxidative stress and mitochondrial dysfunction. Oxidative stress causes excessive levels of reactive oxygen and reactive nitrogen in the mitochondria, exacerbating abnormal mitochondrial function. This ultimately leads to the release of apoptosis-inducing factors and the pro-apoptotic signaling protein cyt c, which then activates the caspase cascade or other apoptotic proteins to form apoptotic vesicles, ultimately leading to neuronal apoptosis. HPA: hypothalamic-pituitary-adrenal axis; intestinal endocrine cells; EECs: BBB: blood-brain barrier; CNS: central nervous system; ROS/RNS: reactive oxygen species reactive nitrogen species.

Oxidative stress and neuroinflammation are two key factors that aggravate nerve injury. In addition, abnormal protein aggregation, excitotoxicity, and neuronal apoptosis also trigger the occurrence and development of NDs. Furthermore, gut microbiota action also influences the CNS through the gut-brain axis (Figure 2). Advanced evidence suggests that anthocyanins could potentially be used in the clinical treatment of NDs. Anthocyanins have been shown to subside NDs by reducing oxidative stress and the viability of microglia. In addition, it can also prevent lipid peroxidation and inflammation caused by neurotoxins, thereby exerting its effects on the nervous system.

### 2.1. Neuroprotective Effects of Anthocyanins on Neurotoxicities Induced by Oxidative Stress and Neuroinflammatory Response

Oxidative stress is a major contributor to neuronal cell death; its clinical manifestation is mainly found in learning and memory dysfunction. Excitatory amino acid and neurotransmitter metabolism and various causes (such as Aggregation of amyloid-β (Aβ) aggregation, calcium imbalance, etc.) can damage mitochondria, thus producing a large number of reactive oxygen species, causing mitochondrial oxidative stress [17]. Activation of glial cells, especially microglia, leads to local inflammation and the death of many neurons. Inflammatory cytokines such as tumor necrosis factor alpha (TNF-α) and interleukin (IL-Iβ) released from microglia first activate astrocytes; cytokines released from astrocytes activate microglia, thus inducing neural cell apoptosis and producing neuronal inflammation [18]. Excessive amounts of many neurotransmitters will produce excitotoxicity in the nervous system, such as glutamate, 2-amino-3-hydroxy-5-methyl-4-isoxazoliproic acid receptors (AMPA), and kainic acid (KA) receptors, and Ca^2+^ and K^+^ will interact with some neurotransmitters (such as glutamate), thereby inducing mitochondrial dysfunction [19,20].

Apoptosis is a direct cause of neurological disorders, and Aβ, lipopolysaccharide (LPS), and glutamate as toxins can cause neuronal cascade responses, including endoplasmic reticulum stress-mediated apoptosis and mitochondria-dependent apoptotic pathways. On the other hand, Aβ peptides induce the accumulation of unfolded proteins, leading to abnormal protein aggregation and increased intracellular calcium fluxes. A combination of glutamate and lipopolysaccharide then induces neuronal apoptosis. On the other hand, mitochondria release apoptosis-inducing factors and cytochrome c (cyt c) and cysteine-specific proteases (caspases) or other apoptosis-related proteins are activated to form apoptotic vesicles, which ultimately disrupt neuronal apoptosis [21].

Anthocyanins can modulate neurodegeneration by acting directly or indirectly on the nervous system. Anthocyanins extracted from bilberries and blackcurrants have been shown in vitro to reduce menadione-induced ROS production in human SHSY5Y neuroblastoma cells over-expressing APP751, thereby protecting SK-N-SH cells from Aβ-induced cellular toxicities [22]. Anthocyanins can restrain the production of pro-inflammatory mediators in microglia and avoid inflammatory cell damage caused by Aβ, thereby exerting neuroprotective effects. The related research mechanism shows that anthocyanins inhibit nuclear factor-κB (NF-κB) activity, c-Jun N-terminal kinase (JNK) activation, and microglial proliferation to suppress neuroinflammatory responses, suggesting that the NF-κB and JNK could be potential targets of anthocyanins [23]. Ullah, et al. [24] found that anthocyanins could reduce Ca^2+^ dysregulation, ROS accumulation, and AMP-dependent protein kinases in KA-treated mouse hippocampal cell line (HT22) and primary prenatal rat neurons (AMPK) activation and increase in the percentage of apoptotic cells. Song et al. [25] found that C3G not only inhibits the spontaneous aggregation of Aβ_25-35_ at the molecular level, but also effectively inhibits the binding of Aβ_25-35_ to the cell surface. In vitro experiments suggested that C3G suppressed reactive oxygen species formation, cellular apoptosis, cleaved caspase-3 expression, and Bcl-2-related X protein (Bax)/upregulated B-lymphocystoma-2 (Bcl-2) ratio, and increased the expression of cyt c release from mitochondria [26].

### 2.2. The Penetration of Anthocyanins through the Blood-Brain Barrier

The three membranes between blood, brain cells, and cerebrospinal fluid are collectively referred to as the blood-brain barrier (BBB), which maintains the stability of brain tissue, protects neural tissue from toxins and pathogens, and limits the penetration of drug compounds used to treat NDS [27]. The polarity of anthocyanins allows molecules to penetrate the BBB. Anthocyanins are transported to the CNS and their accumulation occurs mainly in brain endothelial cells, brain parenchyma, and many tissues, including the striatum, hippocampus, cerebellum, and cortex. In rodent models, anthocyanin accumulation levels of up to 0.21 nmol/g have been found in brain tissue [28,29], which suggests that anthocyanins can accumulate in the brain through the BBB. The above study shows that anthocyanins and their metabolites can transmit to the BBB to protect the brain. However, some recent studies have also found that anthocyanins and their metabolites have difficulty entering the brain through the BBB by giving oral blackcurrant or strawberry AC extracts to male Wistar rats; anthocyanins are not directly related to the prevention of β-amyloid-induced neurotoxicity [30].

There is bidirectional communication between the gut and the brain. The CNS regulates the intestinal epithelium mainly through the vagus nerve, the immune system, and chemical signals, while the intestinal microbiota and its metabolites act as chemical signals that feed back to the CNS through the BBB or the vagus nerve. Microbial metabolites can disrupt the integrity of the intestinal epithelial barrier, which can be transferred to the circulatory system and cause immune cells to produce pro-inflammatory cytokines, thereby inducing the development of NDs. Additionally, some metabolites and cytokines cross the BBB and act on the CNS.

## 3. Interactions between Anthocyanins and Gut Microbiota

Anthocyanins’ health benefits have been widely reported, particularly in preventing oxidative stress-related diseases such as cardiovascular disease, noncommunicable diseases, and inflammatory diseases. There are currently some reports that anthocyanins can affect the gut microbiota (by absorbing metabolism directly in tissues or organs [31]), thereby modulating immune or neural pathways to affect the CNS. Neuronal activity and cognitive function in the brain may be directly or indirectly influenced by neurotransmitter synthesis pathways in the gut [32]. The gut microbiota primarily produces short-chain fatty acids (SCFAs) and modulates the metabolism of bile acids, as well as some neurotransmitters such as gamma aminobutyric acid (GABA), serotonin (5-HT), glutamate, and dopamine. Although unable to cross the BBB, they can be converted into functional neurotransmitters, including dopamine and norepinephrine [33,34]. These findings suggest that the gut microbiota can alter brain function and cognition, primarily by regulating neurotransmitter synthesis and blood levels to influence neurotransmitter delivery to the brain.

In addition, recent evidence suggests that the health benefits of anthocyanins may also be related to the regulation of the gut microbiota. Anthocyanins are only partially absorbed (up to 35%) in the stomach and small intestine, but they interact with the gut microbiota to produce a large number of metabolites and catabolite products [35]. In the intestinal epithelium, liver, and kidney, anthocyanins absorbed by the body are metabolized to glucuronide, sulfate, and methylation by separate metabolizing enzymes [36]. In turn, the intestinal microbiota is involved in anthocyanin metabolism, vitamin synthesis, and carbohydrate catabolism, accompanied by some enzymes, such as α-L-rhamnosidase, β-D-glucosidase, and β-D-glucuronidase. Phenolic compounds produced by anthocyanin catabolism under the action of intestinal microbiota can also inhibit oxidative stress and inflammatory reaction. There is evidence that these metabolites can activate Nrf2 and reduce intestinal inflammation by affecting MAKP mediated by tak1 and the NF-κB pathway mediated by SphK/S1P [37]. Studies have shown that anthocyanins from *Lycium barbarum* can reduce intestinal inflammation, induce SCFAs production, and inhibit endotoxin production by inhibiting LPS/NF-κB/TLR4 pathway.

### 3.1. Metabolism of Anthocyanins by Gut Microbiota

The body absorbs a small number of anthocyanins ingested through the diet, and the vast majority of the ingested compounds reach the colon (pH 7.4–8), where they interact with the microbiota for biotransformation and metabolism before being absorbed across the intestinal mucosa [31]. In the first phase of anthocyanin bacterial hydrolysis, anthocyanin aglycon is formed, mainly due to the cleavage of its sugar group. In the second phase, anthocyanins are degraded to simple phenolic acids in the small intestine, involving the activity of two bacterial enzymes, specifically α-L-rhamnosidase and β-D-glucosidase [38]. The benefits of anthocyanins are manifested in three main ways: absorption from the upper gastrointestinal tract, direct metabolism by endogenous enzymes, and absorption by the colon of metabolites grown by the action of third world anthocyanins and microbiota.

In assessing bioactivity in vivo, the bioavailability of anthocyanins in the gut microbiota is crucial. The bioactivity of anthocyanins depends on their bioavailability, i.e., the amount of anthocyanin that reaches the systemic circulation, which usually refers to the absorption and metabolism of anthocyanins in the body. Because anthocyanin bioavailability is the foundation of their health-promoting effect, it is critical to investigate anthocyanin absorption, metabolism, biotransformation, and elimination. Anthocyanins have a low bioavailability, with only 5–10% of total polyphenol intake absorbed in the small intestine [39]. It is widely believed that the action of intestinal microbiota can increase not only the bioavailability of dietary phenolics, but their antioxidant activity can be increased about fourfold [40]. Anthocyanins benefit from degradation by colonic microbiota, which contribute to anthocyanin bioavailability and may be responsible for antioxidant activity [38]; inhibit angiotensin-converting enzymes from improving blood pressure; improve glucose metabolism, plasma lipid profile, and inflammation [41].

Anthocyanins have been widely reported to have low bioavailability. However, due to the unstable nature of anthocyanins, their absorption, distribution, metabolism, elimination, complex metabolic pathways, and subsequent large amounts of metabolites have not been fully defined. Major degradation products of delphinidin-3-O-glucoside, peonidin-3-O-glucoside, cyanidin-3-O-glucoside, pelargonidin-3-O-glucoside, and malvidin-3-O-glucoside have been identified as gallic acid, vanillic acid, protocatechuic acid, 4-hydroxybenzoic acid, and syringic acid, respectively [42]. Other metabolites produced by anthocyanin degradation include catechol, pyrogallol, resorcinol, tyrosol, 3-(3’-hydroxyphenyl) propionic acid, dihydrocaffeic acid, and 3-(4’-hydroxyphenyl) lactic acid [43]. Through monocarboxylic acid transport by epithelial cells, these acidic metabolites may be absorbed [31]. Three types of anthocyanins in mulberry trees (Cy-3-O-glucoside, Cy-3-O-ruevoside, Dp-3-O-ruetoside) are metabolized by intestinal microbes to produce aldehyde and phenolic acid metabolites (such as protocateic acid and vanillic acid), and the formation of aldehyde and phenolic acid metabolites is catalyzed by associated enzymes or spontaneous cleavage, resulting in a series of anthocyanin monomer transformation steps [44]. Studies have proved that the main absorption point of anthocyanins is jejunum ileum, and it is suggested that anthocyanins are carriers that transport phenols to the intestine, but this still requires further experimental proof [45].

### 3.2. Modulation of Gut Microbiota by Anthocyanins

There may be a relationship between the intake of anthocyanin-rich foods and the composition of the gut microbiome, including increasing the overall microbial population and the growth of specific microbial substances (Table 1). Different anthocyanins acted on different animals or were treated differently and fermented in vivo or in vitro, with similar effects on gut microbiota, even if the microbiota were of different origins. This can be easily detected by fecal microbiota transplantation or simulated in vitro fermentation. However, these studies mainly focus on the ability of anthocyanins to change intestinal flora and improve inflammation and rarely focus on the role of specific anthocyanins and microbiome in health. 

Anthocynins and their metabolites are known for both promotion and inhibition of intestinal microbiota, and can hinder the proliferation of pathogenic bacteria, both gram-positive (*Bacillus subtilis, Staphylococcus aureus*, and *Listeria monocytogenes)* and gram-negative *(Escherichia coli, Salmonella enterica*, and *Citrobacter freundi* [31]. Anthocyanins can stimulate the growth of beneficial bacteria, such as *Bifidobaterium* spp., *Lactobacillus*, *Enterococcus* spp. in vitro and in humans [62], which are recognized as one of the most important bacterial groups associated with human health, anti-obesity effects, and cholesterol regulation [63]. In vitro microbial cultivations revealed that anthocyanin monomers derived from peonidin and purple sweet potato anthocyanins could promote the growth of *Bifidobacterium bifidum*, *Bifidobacterium adolescentis*, *Bifidobacterium infantis*, and *Lactobacillus acidophilus* while inhibiting the growth of *Staphylococcus aureus* and *Salmonella typhimurium* [55]. Black fungus (*Aronia melanocarpa* (Michx.) Elliot) anthocyanins significantly increased the relative abundance of beneficial bacteria, especially *Bifidobacteria*, and decreased the quantity of harmful bacteria, thus improving the intestinal microecology [64]. A study found that in a mouse model of depression treatment, black rice anthocyanins achieved antidepressant effects by regulating the balance of the intestinal microbiota. The species richness and diversity of the intestinal microbiota were altered, with increased levels of *Firmicutes* and decreased levels of *Bacteroidetes* and *Proteobacteria* [65]. In the dextran sodium sulfate (DSS) murine model of colitis, the intake of anthocyanin-rich purple potatoes inhibits intestinal permeability, elevated mRNA expression of inflammatory interleukins IL-6 and IL-17, and elevated protein levels, as well as the relative abundance of specific pathogenic bacteria such as *Enterobacteriaceae* and the relative abundance of *Akkermansia muciniphila* (*A. muciniphila*) to improve some of the symptoms of inflammatory bowel disease [54].

Anthocyanins can also promote short-chain fatty acids to change in a better direction when they act on the intestinal tract. Recently, Sun, Zhang, Zhu, Lou, and He [52] reported a potential prebiotic effect of anthocyanins on gut microbiota, promoting double-ambiguous effects, including improved probiotics (*Bifidobacterium* and *Lactobacillus*) and SCFAs producers (*Roseburia*, *Faecalibaculum*, and *Parabacteroides*), which improved the colon environment and reorganized microbial structure. In vitro simulation of the digestion and fermentation of *Lycium barbarum* polysaccharide by human gut microbiota revealed that anthocyanins from *L. ruthenicum* interacted with gut microbiota to produce SCFAs. In addition, another study also showed that anthocyanins from *L. ruthenicum* promote the production of SCFAs (as evidenced by the promotion of acetate, propionate, and butyrate formation), demonstrating the dynamic regulation of the intestinal microbiota by anthocyanins [47]. Chen et al. showed that anthocyanins can stimulate the growth of beneficial bacteria (*Bifidobacterium* spp., *Lactobacillus* spp., and *Enterococcus* spp.) and inhibit the growth of *Clostridium histolyticum* perfringens (a potentially harmful bacteria) [66]. Therefore, it is generally accepted that dietary anthocyanins can alter the colonization of the gut microbiota, i.e., it has a prebiotic effect similar to regulating the combined position of the gut microbiota [67]. In addition, membrane and intracellular interactions of compounds also influence anthocyanin activity, such as decreasing the release of LPS molecules from the outer membrane of Gram-negative bacteria [68].

### 3.3. Physiological Activity Related to Modulation of Microbiota

It is well-known that in the human body, the gut microbiota is an important producer of vitamins and essential coenzymes for metabolic reactions. Biotin, cobalamin, folic acid, niacin, pantothenic acid, pyridoxine, riboflavin, and thiamine can all be synthesized by gut microbiota, as can vitamin K2.

In addition, vitamins appear to modulate gastrointestinal health, either by modulating the composition and metabolic activity of the gut microbiome or by affecting the gut barrier and immune system [69]. Similarly, beneficial bacteria can produce SCFAs (e.g., acetate, propionate, butyrate, etc.) and other metabolites (e.g., pyruvate, succinate, lactate, soluble oligosaccharides, gases, etc.), thus maintaining glucose homeostasis and intestinal integrity [70].

Li et al. [71] found that anthocyanin-containing potatoes attenuated colonic epithelial damage caused by colitis and maintained intestinal permeability in mice treated with antibiotics in a mouse model. Anthocyanins are bioactive molecules in the colon that maintain the balance of the intestinal microbiome and, in addition, they have similar mechanisms in the prevention and treatment of cardiovascular disease, osteoporosis, cancer and NDs [72]. As mentioned above, the benefits of anthocyanins are mainly achieved through regulating the homeostasis of the intestinal microbiota and producing some metabolites (SCFAs, etc.). *Bifidobacterium* is the main genus of human gut microbiota, and its abundance is closely related to the total number of gut microbiota [73]. By observing the pregnant rats who ingested dietary anthocyanins, *Lactobacillus* and *Bifidobacterium* were recovered in their mothers, NF-κB signaling pathway was down-regulated, and genomic DNA and ZO-1 gene were significantly expressed in their offspring [74,75].

The ability of anthocyanins to alleviate atherosclerosis may be related to the gut microbiota. A study showed that the adhesion of monocytes to endothelial cells (the first step in the development of atherosclerosis (AS)) can be regulated by anthocyanins, intestinal flora, and their metabolites [76]. Wang et al. [77] showed that, mediated by the gut microbiota and its metabolites, anthocyanins promote reverse cholesterol transport under macrophages and alleviate atherosclerotic lesions. In an 8-week vitamin and high-fat diet-induced rat model, oral administration of purified anthocyanins from *L. ruthenicum* (LRPA) significantly increased HDL-C concentrations and the abundance of *Bifidobacteria, Lactobacillus* spp., while decreasing serum levels of TG, TNF-α, IL-6, and atherogenic index, suggesting that LRPA ameliorates atherosclerosis in rats by remodeling the gut microbial community and regulating signaling pathways of arterial inflammation (NF-κB) and hepatic lipid metabolism (SREBP-2) [78]. Thus, it emphasizes the importance of the interaction between the gut microbiota and anthocyanins. Hence, modulating microbiota by probiotics may be a new area for functional food efforts, which aims to reduce the prevalence of cardiovascular, digestive, and neurological disorders.

## 4. Protective Effect of Anthocyanins on Neurodegenerative Diseases under the Microbial-Gut-Brain Axis System

**Figure 3:**
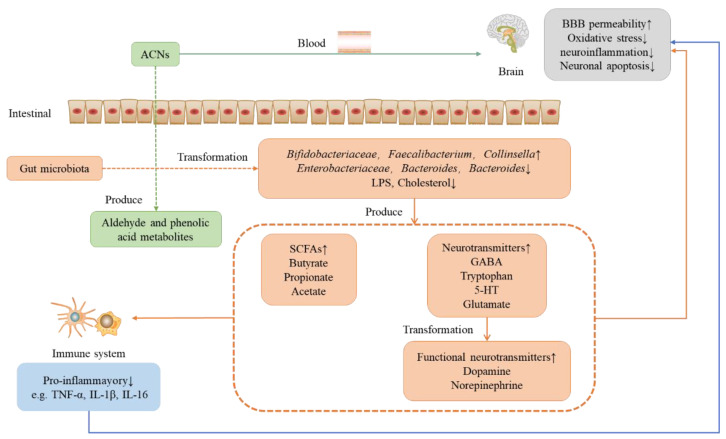
Role of anthocyanins on the nervous system under the microbial-intestinal-brain axis system. ACNS: anthocyanins; LPS: lipopolysaccharide; GABA; gamma aminobutyric acid; 5-HT: serotonin; SCFAs: short-chain fatty acids.

The maturation and development of the human early immune system are related to the gut microbiota, and dysregulation of the microbe-gut-brain axis can cause metabolic diseases and NDs. In addition, the gut microbiota is extensively involved in the synthesis and release of various hormones, active metabolites, neurotransmitters, and other active compounds, in part by regulating the availability of circulating tryptophan, 5-HT, kynurenine, and SCFAs, the permeability of BBB, and the activation of peripheral immune cells and glial cells, thus affecting brain function and host behavior. In addition, altered gut microbiota composition may be involved in the pathogenesis of various neurological diseases, including autism AD, PD, ASD, etc. The gut microbiota interacts with the CNS via multiple parallel and interacting pathways that involve chemical, neuronal, and immune signaling (Figure 3). According to one study, anthocyanins modify host tryptophan metabolism to produce metabolites that control CNS inflammation. As a result, anthocyanins may act as microbe-gut-brain axis mediators to regulate neuroinflammation via gut microbial modulation. In addition, studies have also showed that anthocyanins can regulate the synthesis of certain metabolic products (such as tryptophan, SCFAs, etc.) and certain toxic substances (such as LPS, etc.) by regulating the gut microbiota, thereby increasing the integrity of the BBB and reducing the level of neuroinflammation in nerve cells to achieve the effect of treating NDs.

### 4.1. The Microbiota-Gut-Brain Axis

#### 4.1.1. Neuronal Pathways

Neural pathways are the connections formed by a mix of electric and chemical transmissions within the nervous system access, the direct pathways linking the gut and brain, primarily including the vagus nerve and enteric nervous system (ENS). The vagus nerve (80% of afferent fibers and 20% of efferent fibers) is the main component of the parasympathetic nervous system and the most direct pathway between the intestine and the brain, producing reactions such as adaptive/maladaptiveness, where the refractory responses are mainly manifested in gastrointestinal pathology and NDs [79]. Enteroendocrine cells interact directly with vagus nerve by releasing serotonin (activating 5-HT receptors in afferent fibers of vagus nerve) or gastrointestinal hormones. Significantly, these cells express TLRs, mediating microbial induction. For example, enteroendocrine cells can use LR4 to detect LPS and TLR2 to detect peptidoglycan to identify signals from gram-negative bacteria [80]. Similarly, a matched cohort study from Sweden shows that vagotomy may have a protective effect on PD [81]. However, another study has shown that there is no dependence of gut and brain on vagus nerve in antibacterial therapy, which indicates that there may be an independent neural mechanism in vagus nerve communication [82].

The ENS controls the behavior of the gastrointestinal tract (GI), and is an essential part of autonomic nervous systems, such as intestinal transit and secretion. ENS transmits these signals from the intestinal tract to the brain through the interaction of neurotransmitters (adrenaline, norepinephrine, and acetylcholine), anthocyanins, motor neurons, and the CNS. In addition, the ENS and the CNS are interconnected by the sympathetic and vagus nerve. Gut microflora can regulate the levels of intestinal peptides (such as glucagon-like peptide-1 (GLP-1) and 5-HT), which are secreted by intestinal endocrine cells (EECs) to influence the afferent pathways of vagus nerve, thus regulating intestinal metabolism through the microflora-gut-brain axis [83]. It may also affect the neuroanatomical structure of the ENS, and there is evidence that the immature ENS of germ-free (GF) mice can be normalized by colonization of the gut microbiota [84]. Following electrical stimulation, metabolic compounds of *Bifidobacterium longum* NCC3001 cause a decrease in action potential spikes in myenteric neurons [82]. The gut microbiota not only influences the intestinal neurons but also promotes the settlement and homeostasis of intestinal glial cells in the intestinal mucosa [77].

#### 4.1.2. Neural Immune Pathways

The immune system interacts with the gut microbiota and the CNS. Enterocytes and GALT immune cells such as T cells and dendritic cells (DCs) are important components of the microbe-gut-brain axis, from which some of the microbial metabolites and neurotransmitters released can cross the blood-brain barrier and affect neurons and glial cells in the brain [85]. Microglia can cause neuroinflammation upon external stimulation and are innate immune effector cells of the CNS, while tissue macrophages of the CNS are responsible for maintaining neural networks and repairing injuries [86]. Furthermore, a recent study of GF mice showed that the immune system of CNS can be regulated by gut microbiota by regulating the activation and steady state of microglia [87]. Microbial tryptophan metabolites can influence CNS inflammation by activating astrocyte aryl hydrocarbon receptors [88].

By adjusting intestinal and peripheral immune cells, the gut microbiota can also communicate with the brain, and in some cases, such as brain injury, directly penetrate the BBB, or connect indirectly through afferent fibers and enteric nerve [89]. The abnormality of the gut microbiome leads to the secretion of amyloid and LPS, increases the intestinal permeability, makes other cytokines such as LPS and amyloid enter the intestinal wall, and stimulates TLR4 and other TLRs to produce inflammatory cytokines, thereby increasing the permeability of the BBB, entering the brain tissue, causing neuroinflammation, nerve damage, resulting in neuronal death, and directly affecting the function of the nervous system [90]. Another study reported the discovery of bacterial LPS in postmortem brain lysates from AD patients’ hippocampus and superior temporal neocortex [91].

The gut microbiota can also communicate with the brain by modulating intestinal and peripheral immune cells. The vagus nerve and circumventricular organs are affected by the systemic circulation of immune factors, cytokines, and chemokines. A study has shown that *Bifidobacterium infantis* could correct IL-10/IL-12 from the peripheral blood mononuclear cells of patients with irritable bowel syndrome [92]. Elevated levels of IL-6 in the brain can stimulate the differentiation of astrocytes, hippocampal neurons, and Schwann cells, and regulate autism-like behaviors through synapse formation, dendritic spine development, and defects in the balance of neuronal circuit [93]. Non-inflammatory cytokines also mediate the regulation of brain function by gut microbes. For example, plasma levels of granulocyte colony-stimulating factor were decreased in neonatal mice after exposure to antibiotics, thus reducing the stimulation of the BBB and playing a protective role in NDs [94,95].

#### 4.1.3. Chemical Signaling Pathways

Gut microbiota can produce a spectrum of neurotransmitters, neuroactive compounds, and metabolites, which then regulate the homeostasis of the host CNS directly or indirectly through various chemical signals, such as SCFAs, 5-HT, bile acids, and GABA. *Lactobacillus* and *Bifidobacterium* species, for example, can produce GABA, as *Escherichia*, *Bacillus*, and *Saccharomyces* spp. can produce norepinephrine; *Escherichia*, *Bacillus*, *Lactococcus*, *Lactobacillus*, and *Serratia* can produce dopamine. Studies have shown that there is direct communication between gut microbiota and the hypothalamic-pituitary-adrenal axis (HPA axis, one of the main regulatory factors of human physiological stress response), which affects the CNS. SCFAs could interact with G protein-coupled receptors or histone deacetylase, and act on the brain through humoral, immune, and other pathways, thus affecting inflammation and hormone regulation and interact with the vagus nerve to have an effect on the nervous system [96]. SCFAs are demonstrated to possess immunomodulatory effects, which interact with the CNS to directly activate sympathetic neurons and indirectly affect behavior and neural signals through the BBB [97]. In addition, glial cells in the developing brain can take up SCFAs as a source of energy for cellular metabolism [98].

Similarly, the gut microbiota may play a role in neuropsychiatric disorders by regulating tryptophan levels. Under the action of the gut microbiota, tryptophan produces 5-HT, a neurotransmitter with diverse effects, which can act via both pro-inflammatory and anti-inflammatory mechanisms to affect the intestinal mucosal immunity [99]. Peripheral 5-HT could not cross the BBB, which participates in a variety of human physiological functions by stimulating specific 5-HT receptors. It is an important gastrointestinal signaling molecule that transmits signals from the intestine to intrinsic or extrinsic neurons, thereby influencing intestinal peristalsis, secretory reflexes, and absorption of nutrients. However, we still have no way of knowing the mechanism by which gut-derived 5-hydroxytryptamine affects the CNS [99]. GABA is a major inhibitory neurotransmitter, and GABA secretion by gut microbes may be influenced by intestinal pH, with anaphylactic Bacteroides showing the strongest potential for GABA production in the pH range of the human intestine [100]. Microbial production of GABA in the testis may be involved in the gut-brain connection via the intestinal enteroendocrine pathway and the neuroimmune pathway [101].

The HPA axis is a complex two-way communication network among hypothalamus, pituitary gland and adrenal gland, and regulates various bodily functions. It acts through a series of endocrine steps that lead to the production of cortisol (which inhibits the production of pro-inflammatory factors), which, together with the endocrine and its metabolites (such as adrenaline and norepinephrine), act on the body to maintain endocrine balance [102]. Gut microbiota directly act on the HPA axis to regulate stress and human endocrine, and maintain body homeostasis. HPA axis can also act on the intestinal tract, change the composition of gut microbiota, increase intestinal permeability, promote the infiltration of microbial metabolites into the blood, and produce inflammation [103]. GF mice showed significantly higher HPA axis activity under restrictive stress compared to specific pathogen-free (SPF) mice [104].

### 4.2. The Role of the Microbiota-Gut-Brain Axis in Neuropsychiatric Disorders

Past studies have revealed that the microbial-gut-brain axis is a key pathway for the involvement of the gut microbiota in NDs (Table 2).

#### 4.2.1. Alzheimer Disease

AD is a form of dementia with memory loss and failure to develop normal daily life and behavior. It is the most common form of dementia in the elderly [116]. AD is a NDs caused by the aggregation of Aβ peptides leading to the loss of neurons. Similar to the CNS, the gut microbiota is a source of amyloid. There is growing evidence that probiotics and their metabolites can work through the immune pathway in order to improve neurological disorders. Recently, a study showed that AD’s patients with a combination of probiotics containing Bacillus *B. longum* and *Lactobacillus* spp. had improved cognitive function and metabolic status [117]. Taxa in the fecal microbiome of elderly patients with AD that cause the abundance of pro-inflammatory conditions is higher, which is associated with the production of Aβ. In a mouse model of AD, probiotics increased antioxidant and neuroprotective effects via the SIRT1 pathway, while attenuating cognitive impairment, brain damage, Aβ aggregation, and neuronal protein breakdown levels [106]. The combined effect of probiotics and selenium kawaii improved cognitive function (i.e., higher Mini-Mental State Examination scores) and slowed inflammation and oxidative stress levels, compared to AD’s patients in the selenium-only or placebo groups [105]. Overall, probiotics have potential efficacy to improve cognitive function in both AD patients and healthy populations.

In fact, loss of function of the BBB may also lead to the onset of AD, for example, the gut microbiota produce endotoxin to destroy BBB integrity, thus infiltrating inflammatory factors and microbial-derived metabolites (phenylalanine or isoleucine) to produce the pro-inflammatory effect [118]. It is well-known that age is the greatest risk factor for AD. With age, the permeability of BBB and the integrity of the gastrointestinal epithelium decrease, while gut microbiota homeostasis shifts in an unfavorable direction [119].

#### 4.2.2. Parkinson’s Disease

PD, known as tremor paralysis, is characterized by motor and non-motor symptoms. Gastrointestinal dysfunction is prevalent in PD patients, the initial symptom seen before motor symptoms. Some scholars believe gut microbiota plays a key function in the aggregation of beta-synaptic nuclear proteins and neuroinflammation [120]. During PD, the first and most common nerves affected by alphasynuclein pathology are the enteric and parasympathetic nerves, respectively. Recent research has found that, in the typical pathological changes in PD, the α-Syn in Lewy (Protein aggregate consisting of multiple proteins such as α-synuclein-positive) are expressed in enteric neurons and enteroendocrine. Mouse experiments show that-Syn can be transported across the BBB to the brain, transported from the intestinal mucosa to the CNS, and infusion of Syn FPP into rodent gut trace findings that the risk of PD can be reduced by cutting off the vagus nerve, which blocks its transmission pathways [81,120]. Compared with healthy individuals, changes in the gut microbiota of the PD patients are less *Brlautia* and *Rosaceae*, less *faecobacterium*, and increased *Lactobacillaceae*, *Amancella*, and *Bifidobacterium* [108]. Animal experiments show the movement of PD model mice undergoing fecal transplantation of healthy mice. With improved function, increased striatal neurotransmitters, and decreased levels of neuroinflammation [121]. Another study showed dysregulation of gut microbiota homeostasis and reduced serum LPS binding protein levels in PD’s patients [122]. In a placebo-controlled clinical trial, the investigators found an increase in enzymatic defenses and a decrease in levels of high-sensitivity C-reactive protein and oxidative damage in PD’s patients treated with probiotics (*Lactobacillus acidophilus*, *B. bififidum*, *L. reuteri*, and *Lactobacillus fermentum*) [107].

Therefore, regulating the gut microbiota and improving the function of the gut microbiota-gut-brain axis may be important for the prevention and treatment of Parkinson’s disease.

#### 4.2.3. Autism Spectrum Disorder

ASD is characterized explicitly by limitations and repetition of personal interests or activities, deficits in language learning, and social skills [123]. However, the vast majority of ASD patients, especially children, are accompanied by gastrointestinal disease such as diarrhea and constipation. Several studies have demonstrated the role of GI microbiota in ASD symptomatology [124]. While genes generally cause ASD, environmental factors can also contribute to the development of similar conditions, such as anxiety [123]. More and more evidence has revealed that the gut microbiota of patients with ASD and metabolites (including neurotransmitters) differ from healthy controls [124]. A comparison of stools from 58 ASD patients and 39 normal children revealed that the ASD patients had higher levels of *Lactobacillus* and lower levels of SCFAs (such as acetate, propionate, and valerate) and *Bifidobacteria* [125]. Numerous studies have shown that probiotics positively affect ASD symptoms, improving neurobehavioral symptoms such as anxiety or inattention and/or gastrointestinal symptoms [103]. Fecal microbiota transplantation (FMT) resulted in an approximate 80% reduction in GI symptom scale scores in patients with ASD, and clinical evaluation showed that behavioral ASD symptoms improved and were maintained for at least 8 weeks [109]. The microbial-gut-brain axis of ASD has much to be discovered, especially fecal microbiota transplantation (FMT), and is promising in the treatment of gastrointestinal symptoms in ASD. A recent study showed that oral *Lactobacillus rhamnosus* (JB-1) promotes increased functional brain metabolites such as GABA, N-acetylaspartate, and glutamate, and one step proved that microorganisms can affect brain activity through metabolic routes [126]. Thus, it is observed that the gut microbiota can influence ASD behavior by producing neuroactive metabolites to regulate the excitation-inhibition balance in the brain.

#### 4.2.4. Anxiety and Depression

Anxiety often accompanies mental illness and various physical illnesses, especially among stress-related illnesses. Depression is low mood and/or loss of interest or fun in daily activities for at least 2 weeks, and also includes symptoms such as depression, worthlessness and/or despair, decreased energy, decreased appetite, sleep disturbances, altered psychomotor function, and suicidality and/or action [127]. Major depressive disorder (MDD) is one of the leading causes of disability, with symptoms including decreased appetite and sleep, irritability, poor concentration, and depressed mood [128]. Chronic GIT can lead to responsiveness to stress and MDD in the body [129]. However, many antidepressants are prone to drug resistance, and more scholars are focusing on the intestinal microbiota.

Numerous studies have demonstrated that anxiety is closely related to intestinal microbiota dysbiosis. Behavioral and stress tests on microbiota were conducted on GF zebrafish larvae and found that motor and anxiety behaviors improved with probiotic treatment [130]. Naseribafrouei et al. found that disturbances in the intestinal-brain axis are associated with the pathophysiology of depression [131].

In a study by Jiang et al., by comparing the 16S rRNA sequences of fecal bacteria from 37 depressed patients, it was found that lower levels of anaphylactic bacteria translated to higher levels of Arthrobacter and Enterobacter [132]. In addition, in a placebo-controlled clinical trial, including 40 patients with MDD, their depression was significantly improved by probiotic treatment (*Lactobacillus acidophilus, Bacillus casei*, and *Bifidobacterium bifidum*) [111]. *Oscillobacter* spp. is known to produce valeric acid, a compound similar to GABA, which produces valeric acid and binds to the GABA (a) receptor. The decrease in corticosterone and IL-6 levels in the cecum was accompanied by an increase in SCFAs levels and an increase in plasma IL-10 levels, which may explain the anxiolytic and antidepressant properties of *Oscillobacter* spp. [130].

#### 4.2.5. Schizophrenia

SCZ is a mental disorder which manifests as abnormal thinking, perceptual impairment, memory impairment, psychological abnormalities, and emotional expression disorders [133]. The pathogenesis of SCZ is still unclear, and some studies believe that SCZ has abnormalities in neurotransmitter systems such as dopamine, glutamate, and beta-aminobutyric acid (BABA) [134]. Peng et al. found lower glutamate, higher glutamine, and γ-aminobutyric acid in the hippocampus of mice that received SCZ microbiota fecal transplants and displayed schizophrenic-like behavior [112]. The literature also reported that SCZ’s patients exhibit complex gut microbiota changes compared to the healthy group, such as lower levels of *Haemophilus* spp., *Stachybotrys* spp., and *Proteobacteria phylum*, and higher levels of anaerobic *bacteria* spp. [135]. In addition, both anxiety and depressive symptoms in SCZ’s patients can be effectively improved by probiotic therapy. It was found that intestinal discomfort caused by *C. albicans* was more pronounced in men, and probiotic treatment significantly reduced *C. albicans* antibody in men, but not in women [112]. However, in a randomized, placebo-controlled trial of SCZ-assisted probiotics, the patient’s stomach bowel function improved, but the severity of psychiatric symptoms did not change [113]. Differences in data on altered gut microbiota in SCZ may be interfered with by other external factors, requiring further experimental studies and more clinical trials to improve the treatment of such diseases.

#### 4.2.6. Bipolar Disorder

BD is a complex disorder involving mood disorders, neuropsychological deficits, and disorders of the immune system if severe enough, which can lead to mania. BD patients are at a high risk of suicide, and it is one of the leading causes of disability worldwide. The pathogenesis of BD is still not fully known, but genetics and environment are important influencing factors.

Changes in flavonoid levels could play a role in BD. Coello et al. [136] showed that the abundance of Flavonifractor became higher in patients with BD. A study by Painold et al. reported higher levels of *Actinobacteria* and *Coriobacteria* in BD’s patients compared to healthy controls [114]. The results of Lu et al. [115] revealed that BD patients had a lower intermediate level in Enterobacteriaceae, which suggests that the homeostasis of the gut microbiota and brain function are altered in BD patients. A recent study found that probiotic clinical interventions with Lactobacillus and Bifidobacterium lactis strains resulted in significantly lower readmission rates in patients with mania, with a particularly pronounced relief of the inflammatory response [136].

### 4.3. The Protective Effect of Anthocyanins on the Nervous System under the Microbial-Entero-Brain Axis System

Accumulating evidence suggests that for NDs, partly because of oxidative stress and neuroinflammation, etc., neuroinflammatory processes are modulated by peripheral inflammation, particularly from the gut microbiota. There is evidence that NDS may have originated in the gut. In addition, changes in gut microbiota homeostasis are also closely related to the anti-inflammatory properties of anthocyanins. It regulates the gut microbiome through the microbial-gut-brain axis and acts as a preventive role in NDs. Under the microbial-gut-brain axis system, the effect of anthocyanins on the nervous system is shown in Figure 3.

Anthocyanins may be slowing neurodegeneration through their regulation of the gut microbiota. An analysis revealed that consuming blackberry anthocyanin-rich extract (BE) decreased TCK-1 expression in the hippocampus of rats, while expanding *Pseudofavonifractor* and *Sporobacter* modulated gut microbiota composition. BE alters CNS inflammation through the use of tryptophan microbial metabolites. In a study, tryptophan (one of the identified metabolites) was decreased in the groups processed by BE, which suggests that BE, gut microbiota, and tryptophan metabolism are closely related [137]. Tryptophan is the precursor of serotonin and kynurenine, which, as an essential amino acid, can affect the CNS’s function through the microbiome-gut-brain axis [138]. The vast majority of tryptophan is metabolized via the kynurenine pathway, about 90% [139]. Tryptophan and kynulic acid (anti-inflammatory properties) can be converted into each other, which could regulate the glutamate level [140]. The gut microbiome modulates serum and urinary levels of kynurenine quinolinic acid, which may alter the state of the CNS [141]. In addition, tryptophan has been shown to regulate neurotransmitter synthesis and maintain the integrity of the intestinal barrier by promoting serotonin synthesis and indole production in the intestine [142]. The gut microbiome can regulate levels of serotonin, while at the same time regulating some gastrointestinal dysfunction due to intestinal axial imbalances and neuro-endocrine-immune stimulation [143]. In summary, anthocyanins not only influence host tryptophan metabolism, but they also produce metabolites that regulate CNS inflammation.

After absorption in the gastrointestinal tract, anthocyanins can also affect brain structures by crossing the BBB and localizing to different areas [30]. Anthocyanins enter the colon and are hydrolyzed by the microbiome; the health benefits of anthocyanins are mainly attributed to their interaction with the intestinal microbiota. Upon entering the colon, anthocyanins are hydrolyzed by intestinal microbes, producing colonic metabolites that, in turn, act on anthocyanins. Positive changes occur in the gut microbiome through the intake of anthocyanin-rich foods, such as increased growth of beneficial bacteria (Bifidobacterium spp.) and increased growth of specific microbial metabolites (SCFAs) [144]. In vitro experiments have shown that the gut microbiota can produce neurotransmitters under certain conditions, such as *Lactobacillus* and *Bifidobacterium* secreting GABA and *Candida*, *Streptococcus*, *Escherichia coli*, and *Enterococcus* secreting 5-HT [145]. It is possible that consuming foods rich in anthocyanins could reduce total plasma cholesterol, TNF-α, and the concentration of LPS, as well as lead to a greater production of fecal SCFAs and favorably modulate the gut microbiota [146]. SCFAs are one of the key factors in the “gut-brain axis” that regulate many host processes, particularly in the CNS, such as mitochondrial function, gene expression, cell-cell interaction, microglia activation, and neurotransmitter synthesis and release [147]. SCFAs produced under the action of the gut microbiota stimulate the intestinal nerves and acts as a protector of the CNS by metabolically regulating immune cells [148]. In addition, it can cross the BBB and interact with microglia to reduce the inflammatory response of microglia [149]. GF mice showed more severe BBB permeability than their SPF counterparts due to reduced expression of endothelial tight junction proteins in the BBB [150]. Anthocyanins-rich supplements (brand name Metabolaid^®^) have been shown to increase the abundance of the main genera of the gut microbiota, *Bifidobacterium*, *Blautia*, and *Faecalibacterium,* as well as other minor genera like *Prevotella* and *Akkermansia*, promote an increase in SCFAs, particularly butyric acid [151]. Subsequent studies revealed that one of the key factors in maintaining the integrity of the BBB is the production of butyrate by gut bacteria [145]. Although the mechanisms are unknown, anthocyanidins gut microbiota metabolites can cross the BBB and reach different brain regions. However, it is worth noting that anthocyanidins have a prebiotic effect that helps regulate gut microbiota and its metabolites, thereby inducing a protective nervous system effect.

Between the “gut-brain axis”, the gut microbiota plays a key role in bi-directional communication, i.e., forming the “microbe-gut-brain axis”. The disorders of the gut microbiota are more associated with a variety of NDs. Cerebral ischemia due to acute brain injury leads to dysregulation of the gut microbiota, which leads to altered species diversity of the gut microbiota and increased intestinal barrier permeability. In addition, dysfunction and neuroinflammation in ICH mice are ameliorated by reprocessing of the healthy microbiota treatment [152]. A recent study found that using anthocyanin-rich blueberries and FMT treatments could modulate the gut microbiota of obese mice to improve metabolic health [153]. Many factors contribute to the homeostasis of the gut microbiota, such as oxidative stress, intestinal pathogens, and chemical exposure. LPS secreted by dysbiosis of the gut microbiota that exaggerate AD pathology, and if microglia receptors (TLR2, TLR4, and CD14) bind to LPS on the bacterial surface, NF-κB transcription factors are activated. Activation of NF-κB produces pro-inflammatory cytokines, which trigger a neuroinflammatory response and produce various NDs [91,154]. In addition, it may disrupt the BBB and increase the level of LPS, which activates the production of cytokines by dendritic cells in the intestinal barrier, such as the production of Aβ peptides in the presence of Salmonella, E. coli, and Citrobacter, etc. These signals may be transmitted to parasympathetic neurons of the vagus and enter the nervous system through retrograde axon transport, thus contributing to peripheral and systemic inflammation [155,156]. In an animal model of LPS-induced neurotoxicity (24 mg/kg/day for 2 weeks: 1 week before LPS and 1 week of LPS), anthocyanin treatment increased the survival of p-Akt and p-GSK3β proteins [23]. In a rat model treated with LPS, the use of FMT can balance the gut microbiome and produce higher levels of short-chain fatty acids, thus preventing neuroinflammation, reduced gut motility, mental weakness, and impaired gut barrier function [157]. Anthocyanin treatment was found to reduce the relative abundance of the Prevotellaceae family, including the *Prevotella* 9 and *Prevotella* 2 genera, in an LPS-treated weanling model, and to enrich the Lactobacillus family while increasing the Firmicutes/Bacteroidetes ratio, thereby reducing the inflammatory response [158].

In the intestine, anthocyanins react with the gut microbiota to produce aldehyde and phenolic acid metabolites, and the colonization of the gut microbiota is altered (e.g., increased levels of Bifidobacteriaceae, Fasciola, and Corynebacteriaceae and decreased levels of Enterobacteriaceae, Bacteroidetes, and Bacteroidetes), along with the production of microbial metabolites such as SCFAs and neurotransmitters. In the presence of gut microbiota, some neurotransmitters can be converted into functional neurotransmitters that cross the BBB and act on the CNS; SCFAs can also function on the immune system and decrease the levels of pro-inflammatory factors in turn affect the CNS. Tryptophan promotes the synthesis of neurotransmitters in the intestine, especially 5-HT and glutamate, while acetate promotes the permeability of the BBB. In addition, anthocyanins can also act directly on the CNS to prevent and treat NDs.

## 5. Conclusions and Expectations

The increased incidence of NDs poses a threat and a heavy burden to human society. In this study, we reviewed the mechanism of action of anthocyanins on NDs and their interaction with the gut microbiome, and it is shown that anthocyanins can achieve a protective effect on the nervous system under the microbial-intestinal-brain axis system by regulating the intestinal microbiota and through the action of certain metabolites. Previous studies have shown the health benefits of anthocyanins and the gut microbiome, particularly in preventing and treating diseases affecting the elderly, including cardiovascular disease, certain cancers, NDs, and age-related disorders such as bone loss. It has been increasingly demonstrated that anthocyanins regulate the CNS through the gastrointestinal tract or through the immune, neurological, and endocrine systems. It is also worth exploring the mechanism by which anthocyanins pass through the BBB to regulate the microbial-intestinal-brain axis.

Although the microbiome-gut-brain axis has developed rapidly in recent years, it is still in its infancy; there are still some challenges for future development. The underlying mechanism of anthocyanins of different structures for NDs under the microbial-intestinal-brain axis system needs further study, and other structures of anthocyanins may have different targeting molecules and initial mechanisms. The molecular mechanisms of the intricate interaction of anthocyanins and different kinds of gut microbes on the beneficial or pathogenic effects of neurological diseases should be more deeply elaborated. Tackling these challenging questions will help validate new causes of microbial-gut-brain axis-mediated neurological diseases and help explore anthocyanin therapies and other potential treatments.

## Figures and Tables

**Table 1:** Gut microbiome alterations following consumption of anthocyanins rich foods.

Sources	Ac	Subject	Microbiota Sources	Intervention	Microbial Modulation Effects	Ref
*Lycium ruthenicum* Murray Fruit	Pg	in vivo: mice	Male C57BL/6J mice	standard diet, lipid rich food, HFD, and oral P3G (100 mg/kg body weight). fecal microbiota transplantation	↑*Bifidobacteriaceae*, *Helicobacteraceae*, *Deferribacteraceae* ↓*Firmicutes*, *Lactobacillaceae*, *Streptococcaceae*, *Erysipelotrichaceae*	[46]
Black chokeberry (*Aronia melanocarpa* (Michx.) Elliot)	Cy	In vitro	5 healthy volunteers who had not taken antibiotics for at least three months (3 males and 2 females, aged 22 to 28-years-old)	anthocyanin (1 g/L), tea polyphenol (1 g/L) mixed in growth medium 9 mL and fecal slurry suspension 1mL and adjusted in gastrointestinal digestion	↑*Bacteroides*, *Bififidobacterium*, *Blautia*, *Faecalibacterium* ↓*Prevotella*, *Megamonas*, *Escherichia/Shigella*	[47]
Plums Italian red grapes Elderberry fruits	Cy	in vitro	strains	Rogosa, Sharpe broth (mMRS) fermentation by *L. rhamnosus* IMC 501^®^, *L. para-casei* IMC 502^®^, SYNBIO^®^ and *L. plantarum* IMC 509, the fruit extracts (10 g/L) conduct growth studies CP: MRS broth Medium (containing glucose at a concentration of 10 g/L)	↑*Lactobacillus rhamnosus* ↓*Bacillus cereus*; *Staphylococcus aureus*; *Escherichia coli*	[48]
Black raspberry	Cy	In vivo: C57BL/6J mice	C57BL/6J mice (five-week-old, 18–20 g)	Normal diet; contains no anthocyanins diet; BRB anthocyanins in the diet, 3.5 µmol/g (LBA), and 7.0 µmol/g (MBA)	↑*Eubacterium rectale*; *Faecalibacterium prausnitzii*; *Lactobacillus* ↓*Desulfovibrio* spp.; *Enterococcus* spp.	[49]
Red cabbage	Cy	In vitro	Fecal samples were collected from 14 healthy volunteers (4 males and 10 females, aged 17 to 52-years-old)	Fecal suspension (1:10, W/V) was mixed with an extract rich in anthocyanins and sterile medium to simulated gastrointestinal fermentation	↓*Lactobacillus* spp. *Clostridium* spp. *Bacteroides* spp. *Enterococcus* spp*. Enterobacteriaceae*	[50]
Cherries polyphenols	Cy	In vivo In vitro	10 healthy volunteers who had not taken antibiotics for at least 3 months (5 males and 5 females, aged 23 to 30-years-old)	In vivo: consume 8 oz of juice daily for five days In vitro incubations were performed by mimicking gastric, intestine, and colon conditions	↑*Bacteroides*, *Collinsella, Firmicutes*, *Enterobacteriaceae*, *Bilophila*	[51]
Purple sweet potato	Pn	In vitro	strains	/	↑*Bifidobacterium* spp.; *Lactobacillus acidophilus* ↓*Staphylococcus aureus*, *Salmonella typhimurium*	[52]
*Lycium ruthenicum* Murray	Pt	In vitro	Fecal samples were collected from 4 healthy volunteers and 3 IBD patientshealthy volunteers (3 males and 1 female, aged 20 to 27-years-old) IBD patients (2 males and 1 female, aged 31 to 56-years-old).	Fecal samples were treated to obtain fecal suspension and then mixed with autoclave base growth nutrient medium (Mixed with P3G (1.0 g/L), ACN (1.0 g/L), and glucose (10.0 g/L), respectively) for in vitro fermentation	↑*Collinsella, Bifidobacterium, Streptococcus* *Lactobacillus plantarum* ST-III ↓*Escherichia, Shigella*	[53]
Purple Potato	Pt	In vivo: C57BL/6 mice	C57BL/6 mice (male, four-week-old)	AIN-93G diet, DSS diet, DSS + 15% purple potato in standard diet, DSS + 25% purple potato in standard diet	↑*Bififidobacterium* spp., *Lactobacillus* spp., relative abundance of *A. muciniphila*	[54]
Grape	Mv	In vitro	Fecal samples were collected from 3 healthy volunteers who had not taken antibiotics in at least 6 months.	To mimic colon fermentation, fecal content was incorporated in fecal suspension (1:10) malvidin-3-glucoside (20 and 200 mg/L), gallic acid (150 and 1000 mg/L), and enocianin (4850 and 48,500 mg/L), then injected in the vessels containing fecal slurry.	↑*Bifidobacterium* spp. *Lactobacillus-Enterococcus* spp. ↓*Bacteroides* spp. *Clostridium*	[55]
*Vitis Amurensis Rupr* of “Beibinghong”	Mv	In vivo: mice	Feces of experimental male ICR mice	Intraperitoneal injected of normal saline, given D- gal (500 mg/kg bw) once a day, given intragastric administration of malvids anthocyanins and stabilization malvids anthocyanins of 50 mg/kg bw every day, respectively	↑ *Lactobacillus, Alloprevotella* ↓ *Bacteroides, Alistipes*	[56]
Black rice	Cy, Pn	In vitro	strains	/	↑*Bifidobacteria*; *Lactobacillus*	[57]
Purple sweet potato	Cy, Pg	In vitro	Fecal from 8 healthy volunteers (4 males and 4 females, age 25 to 30-years-old)	Anthocyanin samples or fructooligosaccharide (FOS) (prebiotic) were combined at a final concentration of 1% (*w*/*v*) with autoclaved nutrient basal growth medium for in vitro fermentation.	↑*Bifidobacterium* spp.; *Lactobacillus*/ *Enterococcus* spp. ↓*Bacteroides-Prevotella*; *Clostridium histolyticum*	[58]
Anthocyanins supplied by Extrasynthese	Cy, Dp	In vitro	Fecal from 3 healthy volunteers, who had not ingested antibiotics for at least 6 months.	Fecal samples were treated to obtain fecal suspension (10%, *w*/*w*) and then mixed with sterile medium and encapsulated anthocyanins to obtain fecal slurry (20 mg/L). The positive control was added with prebiotics, while the negative control was not.	↑*Bifidobacteria*; *Lactobacilli* ↓*Clostridium histolyticum*	[59]
Red wine: dealcoholized red Wine	Dp, Mv	In vivo (randomized cross-over-controlled trial (three consecutive periods of 20 days each)	9 males, age 45 to 50-years-old	The participants were allowed to drink after 20 days abstaining, DRW (272 mL/d, containing 30 g ethanol), or RW (272 mL/d, containing 30 g ethanol),	↑*Bifidobacterium*; ↓*Enterococcus*, *Eggerthella lenta*	[60]
Blueberry	Mv, Pt	In vitro	Taken fecal content from three participants who have not taken antibiotics for 25 weeks	Fecal samples were treated to obtain fecal suspension (10%, *w*/*v*) and mixed with high pressure growth nutrient medium with or without BA (10.0 g /L) simulated fermentation.	↑*Actinobacteria*, *Alloprevotella*, *Faecalibacterium*, *Bififidobacterium*, *Streptococcus*	[61]

Note: “↑” means increase; “↓” means decrease.

**Table 2:** Characteristics of prior studies investigating the relation between microbiome and neuropsychiatric disorders.

NDs	Subject	Study Design	Species Intervention	Consequences	Ref
AD	AD patients AD selenium probiotic group (n = 27); age 78.56 ± 8.0 AD selenium group (n = 26); age 78.86 ± 10.2 AD placebo group (n = 26); age 76.26 ± 8.1	Randomized, double blind, and controlled clinical trial Microbial interventions	Microbial interventions: *L. acidophilus*, *B. bififidum*, and *B. longum* (2 × 10^9^ CFU/g)	Patients with AD had improved cognitive function (increased MMSE scores) and good results for specific inflammatory and oxidative stress markers such as TAC and GSH	[105]
AD	Three transgenic mouse models of AD: B6;129-Psen1tm1Mpm Tg (APPSwe, tauP301L) 1Lfa/J (namely, 3xTg-AD) and control wild-type animals.	Analysis of RARβ Acetylation Redox Enzyme Activity Assays Western Blotting Analyses Oxyblot Analysis	SLAB51: Formulation of lactic acid bacteria and Hypertrendella	SLAB51 Activates SIRT1 Pathway in AD Mice, increases antioxidant enzyme activity-SOD, GPX, and reduces peroxidation levels of proteins and lipids	[106]
PD	PD patients PD probiotic group (n = 30); age 68.26 ± 7.8 PD placebo group (n = 30); age 67.76 ± 10.2	Randomized, double-blind, placebo-controlled trialMicrobial interventions	Microbial interventions: *L. acidophilus*, *B. bififidum*, *L. reuteri,* and *L. fermentum* (each 2 × 10^9^ CFU/g)	PD Patients have a more beneficial MDS-UPDRS score.	[107]
PD	PD patients at different stages (n = 237) PSP (n = 22) MSA (n = 22) HC (n = 113)	Prospective observational case-control studies 16S rRNA gene sequencing	/	PD (compared to HC): Lower levels of Lachnospiraceae PSP (compared to PD): Lactobacillaceae similar, Streptococcaceae were reduced. MSA: Lachnospiraceae were not lower, Prevotellaceae were reduced	[108]
ASD	ASD patients (n = 30); age 5 to 9-years-old	Prospective, open-label study. Microbial interventions. Evaluation of GI flora using quantitative real-time PCR	Microbial interventions: *Lactobacillus acidophilus*, *Lactobacillus rhamnosus*, and *Bifidobacteria longum* (1 × 10^8^ CFU/g)	In terms of the severity of autism (assessed by ATEC) and gastrointestinal symptoms (assessed by 6-GSI), the colony counts at the level of Bifidobacterium and lactic acid bacteria increased, weight loss decreased, autism levels improved	[103]
ASD	ASD patients (n = 18); age 7 to 16-years-old	Open-label clinical trial FMT (Two weeks of antibiotic therapy) and MTT treatment	/	After treatment, the gastrointestinal symptoms of patients were reduced by 80% and ASD behavior symptoms were significantly improved. MTT: ↑*Bifidobacterium*, *Prevotella*, *Desulfovibrio*. All remained improved 8 weeks after treatment ended.	[109]
Anxiety/Depression	Male C57BL/6J mice	Prebiotics (fruco-oligosaccharides (Fos) and galacto-oligosaccharides (Gos)) administrationFos, Gos, Fos+Gos for 3 weeks	/	Antidepressant and anxiolytic effect. Reduced stress-induced corticosterone release and promotes the normalization of the intestinal microbiota	[110]
Anxiety/Depression	160 participants:110 MDD patients, 27 healthy controls, and 23 psychiatric controls. Participants (68 males and 92 females, age 20.0 ± 1.9, 29% taking SSRIs) MDD patients (60% in acute episode)	Longitudinal study underwent a diagnostic evaluation and provided a stool sample. 16S rRNA gene sequencing	/	Whether it is in the period of major depression or remission, and whether it is using SSRIs, it is not related to different bacterial composition.	[111]
SCZ	Human studies: SCZ patients’ group (n = 63) HC group (n = 69) no significant differences in age, sex, or body mass index Animal studies: GF mice	Human studies: provided a stool sample and 16S rRNA gene sequencing. Animal studies: FMT experiments, behavioral tests, Y-maze	/	The reduction in microbial diversity in SCZ patients, which at the same time led to lower glutamic acid and higher glutamine and GABA in mice, suggests that the SCZ microbiome itself can alter neurochemical and neurological function in ways that may be relevant to SCZ pathology	[112]
SCZ	SCZ probiotic group (n = 30), 22 males and 8 females, age 44.66 ± 11.4; SCZ placebo group (n = 26), 15 males and 11 females, age 48.11 ± 9.6	Longitudinal, double blinded, and placebo controlled	Microbiota intervention	It was found that there was a relationship between candida albicans seropositivity and more serious positive mental symptoms. However, administration of probiotics can normalize the antibody levels of candida albicans.	[113]
BD	BD patients’ group (n = 32), 18 males and 14 females, age 41.3 ± 14.7 HC group (n = 10)	Provided a stool sample and 16S rRNA gene	/	An inverse relationship was observed between illness period and micro alpha diversity (r = 0.408, *p* = 0.07) HC (n = 10)	[114]
BD	BD patients’ group (n = 36); HC group (n = 27) no significant differences in age, sex, or body mass index	Rating Scale Assessment for Mood Symptoms Fecal Bacterial Population Determination	BD subjects were treated with quetiapine (300 mg/d) for four weeks.	Quetiapine treatment was effective for depression. After treatment, MARS score decreased, while the levels of rectal *eubacteria*, *Bifidobacterium*, and B/E increased.	[115]

## Data Availability

Not applicable.
